# The Marketplace for New Caries Management Products: Dental Caries Detection and Caries Management by Risk Assessment

**DOI:** 10.1186/1472-6831-6-S1-S6

**Published:** 2006-06-15

**Authors:** Joel H Berg

**Affiliations:** 1 Professor and Chair, Department of Pediatric Dentistry, The University of Washington School of Dentistry, Seattle, WA 98195-7136, USA

## Abstract

The number of new technologies emerging each year in the realm of dental caries management is growing at an exponential rate. Examining the patent literature, one can see that this growth rate will likely continue, with the outcome that dentistry will see an expanded growth in managing dental caries by risk assessment with medicinal therapeutic interventions. Restorative dentistry solutions, treating the results of dental caries, will continue to grow, while technologies to identify the caries process at its earliest stages will soon invade practices everywhere. The most interesting aspect of these changes will be how industry responds to the inexorable, yet slow change in dental professional demand for these new technologies, while trying to be the "first to market" within the various categories of this business opportunity. This paper will take a close look at how businesses with the core competence to be key players in this emerging growth category will assess the marketplace, and match up their business interests with the changing needs of the dental profession. The paper will also address the strategic planning and business processes that the dental industry will undertake to bring new technologies to market, and how these technologies will be positioned to health care professionals and consumers. The results of the key interactions between industry and the dental profession will determine the extent to which dental caries is managed as a disease, in addition to being managed by surgical restorative interventions.

## Introduction

Although dental caries is the most prevalent infectious disease in humans, affecting 97% of the population in their lifetimes, we primarily treat the effects of dental caries, and not the disease itself. Most restorative dentistry, most prosthodontics in adults, and most endodontics are related to the results of dental caries, not the disease process itself. We are, in general, limited to surgical restorative intervention because we have historically lacked clinical caries detection tools sensitive enough to see a caries lesion so early that we can treat it with medicinal therapeutic approaches. Such remineralization techniques are well established scientifically *in vitro*, but have escaped routine clinical use because of the void of early detection methods that are clinically feasible.

In addition, when it comes to reducing the risk of caries within populations, or groups of patients within a practice, we have, in general, provided empirical standardized recommendations, such as "brush and floss" and "use fluoride toothpaste." Although such methods of intervention to reduce caries risk are extremely effective within populations at risk, these routine measures do not target individual patients who may be at much greater than average risk. New ways of thinking combined with new technologies will dramatically change the way we deal with dental caries. Managing the disease process by mitigating risk instead of identifying the disease at a later stage requiring surgical restorative intervention will soon become the norm. To date, the dental industry has not focused much attention on the disease – dental caries – but has focused its attention on the results of the disease – cavities.

The "gross domestic product" of dentistry stands at around $80 billion per year in the US. The dental business in the US is growing at a rate greater than the economy as a whole. The majority of dental expenditures (80 percent) are via professional services, with only 12 percent of expenditures for consumer products (toothpastes, mouthrinses, etc.) and 8 percent for professional products (consumables and equipment used by dental practices in delivering care). When examining the $80 billion from the perspective of the reason for the expenditure, it is apparent from various industry reports that about 60% of the total expenditure, or approximately $48 billion dollars per year in the US, is spent on treating the "effects" or "results" of dental caries. As noted above, the vast majority of caries expenditures (most restorative dentistry) do not treat the disease but provide surgical repair for the damage done by the disease. The reason is that to date dentists have not possessed the necessary tools to detect the caries process on a site-specific basis until such a late stage that surgical intervention is the only available approach.

Indeed, the caries detection devices most of us are using today are extremely insensitive. Visual examination, using explorer and/or mirror usually can identify caries lesions only when restorative intervention is needed. Radiography is also extremely insensitive. Dentists can see caries lesions interproximally on bitewing radiographs only when they are at least halfway through the enamel. This limitation causes us to miss many lesions at the earliest stages, the time when remineralization techniques might be effective. Transillumination to identify caries lesions in anterior teeth has been used for a long time; however, such detection is limited to identifying lesions that are already extensive in their progress through the enamel on the way to cavitation.

In summary, we have been historically unable to detect caries lesions early and subcategorize our patients into various risk categories. The changes that have happened and that will transpire in this arena will change dentistry perhaps more than any change to date.

### Sensitivity, Specificity, and Reliability

When assessing new caries detection tools, one must evaluate them based on several important criteria, including sensitivity and specificity. I will not provide a complicated mathematical definition of these terms, but will rather provide a working definition that is important in the way clinicians should think about such tools.

Sensitivity refers to the ability of the tool or device to identify the presence of the condition when it does indeed exist. In other words, are there any false negatives? By this measure, as mentioned above, we know that all of the tools that have historically been available to us are extremely insensitive. Included in this list of insensitive dental caries detection tools are (1) visual examination, (2) radiography, and (3) unaided transillumination. The absence of highly sensitive devices in the marketplace for caries detection has perpetuated the scenario wherein surgical restorative intervention is general the only option.

Specificity refers to the ability of the tool or device to be accurate in its identification of a condition when it detects such a condition. In other words, are there false positives? There are two parts of the specificity equation to think about. The first: Is what I have detected indeed what I believe it to be? If a radiograph or visual examination detects what is believed to be a caries lesion, how certain can one be that what is detected is indeed a caries lesion?

The second important part of specificity, which will become even more important to us as we begin to detect caries lesions at a very early stage, is: Will the detected lesion progress if untreated? This is, perhaps, the more difficult challenge. The earlier we detect caries lesions, the greater the risk that we will detect lesions that may not have progressed to a stage requiring surgical restorative intervention. The natural compensatory remineralization process might allow routine "reversal" of very small lesions by "naturally occurring" remineralization. This second aspect of specificity – will the lesion progress if untreated? – is important to understand. Having said this, the author and most experts in the area are not too concerned about employing remineralization techniques for early detected small lesions, even at the risk of treating some that may not have progressed if untreated. The greater concern is if these early lesions are treated via surgical restorative interventions when they either (1) might not progress at all or, (2) might be (in the near future) treatable with medicinal remineralization approaches.

The marketplace will ultimately not be receptive to these newer highly sensitive tools, even with increased specificity, unless the appropriate compensation systems are in place. Third party payors are not currently positioned to reimburse for remineralization therapies; in fact, they do not yet reimburse for detection techniques that might obviate the need for radiographs (currently, a billable service).

### Risk Assessment Tools

There are a variety of means available today to assess the risk of patients for dental caries. These tools can be grouped into two distinct areas. First, those that gather historical and environmental data and determine a risk level based on this data; second, those that employ various forms of technology by assessing one or more distinct outcome measures as validated determinants of risk [1,2].

#### History and Environment Tools

Featherstone [3-5] and others have developed a risk assessment tool that offers separate sections for adults and children. This tool includes historical factors, environmental factors, and uses some technology to assess such factors as bacterial counts and salivary flow rates. There is little information validating the use of bacterial counts, salivary flow rates, and buffering capacities in children. The American Academy of Pediatric Dentistry (AAPD) has recently published a caries assessment tool (CAT) [6] that allows the clinician to assign a relative risk to a child by virtue of historical and environmental data collection. The greatest risk factor for caries is a history of caries. Even if a child has had a single surface caries lesion, the risk for future caries is dramatically increased [7]. Additionally, a history of caries in the family, in particular in the mother, will increase the caries risk in the child. This CAT is very useful in caries risk assessment for children, although it does require several minutes of time in the office to gather the needed data.

The marketplace of dentistry that includes the patient, the dental professional, and third party payors must convene regular discussions to allow payment for important services that will lead to this "new kind of dentistry" in which patients are offered highly valuable screening services such as risk assessment and subsequent medicinal and other therapeutic interventions short of surgical restorative intervention. Although it is assumed that most patients would prefer to receive earlier non-surgical treatments, these treatments will not be universally available until compensation means are set in place.

### Technology Assessment Tools

Besides looking at the environmental conditions and the history of the patient as a means to determine caries risk, there are a multitude of technologies in various stages of development which will aid in predicting future caries. Over the next several years, outcomes validation data will emerge to determine which of these technologies or collections of technologies will have greatest predictive value.

#### Acid Production Detection

Although there is no technological magic bullet in predicting caries risk in children, technologies that allow the measurement of "acid production potential" appear quite promising. Regardless of the quantity or strain of organisms within a plaque biofilm, the biofilm must be capable of producing acid upon being challenged with sucrose in order for the caries process to progress. Therefore, any device that uses technology to assess the acid production potential of the biofilm as an "*in vitro *diagnostic" might be quite useful in dentistry for children.

A product referred to as "Cariostat" (Dentsply Sankin, Japan), available in Japan but not in the US, resides in this category. Shimono and his colleagues at Okayama University have studied this interesting risk assessment tool and have been able to reliably predict caries risk as measured by the decayed and filled surfaces (df) outcome measure [8-10]. The Cariostat test has reliably predicted caries experience in the short term in toddlers, and in the long term by sampling as early as age 3 and predicting df outcomes as long-term as age 10. Additionally, Shimono's group has shown that aggressive intervention within a Cariostat elicited high-risk group can prevent subsequent caries experience in such high risk kids.

Other technologies are currently being developed in the category of acid production potential. When technologies within this category are validated via caries outcome measures, these tools may be extremely useful in a variety of environments not limited to dental offices. Pediatricians are now required to perform an oral health assessment at 6 months of age, [11] given that they see children so often at a very young age (15 times or more before age 3). If technology can provide them with a rapid-screening tool that is reliable, then it might easily be determined which of the millions of children they see each year need more immediate referral for intervention and prevention of dental caries.

#### QLF (Quantitative Light-Induced Fluorescence

Inspektor Research Systems (Amsterdam) offers another very interesting technology in the early detection of dental caries. This device is currently marketed in the US by OMNII Oral Pharmaceuticals. It is being used in a variety of laboratory and clinical trials to identify caries lesions at a very early stage. Because of its ability to detect caries early and on a lesion-specific basis, it will likely precipitate the development of new drugs for early stage intervention, prior to the need for restorative treatment. The QLF device transmits light to evoke fluorescence. A collection component receives the fluoresced tooth data and calculates demineralization. In the case of QLF, the device uses a halogen light within the visible frequency and "scans" the tooth surface, providing an image of the entire tooth. The intellectual property of this device resides in its analytic software that can provide a very detailed analysis of the demineralization level of very early lesions, and can also "superimpose" scans over previous scans of the same lesion in order to identify the effects of treatment. When perfected clinically, this device may be exceptionally important to clinicians in the early detection and individualized management of caries lesions with remineralization approaches. Studies have already shown that QLF might be useful in monitoring lesions while engaging in remineralization therapies.

### Caries Management with Fluoride Interventions

Other papers in this publication deal with fluoride and other medicinal interventions for caries management. Because we now live in a world where caries can be detected before restoration is warranted, the obvious question that arises after early detection is, "Now what do I do"?

A variety of intervention measures are currently available, and many more are being developed [12-14]. Many of these interventions are using products we have in our possession, such as fluorides in various forms. Fluoride varnish, a new member of the preventive armamentarium, might be the precursor to soon-to-be-available varnish type interventions employing other agents instead of, or in addition to, fluoride. We may also see the development of lesion-specific treatments that are professionally applied, in combination with individually tailored home care programs for the parents and the child [15-25]. By detecting caries lesions and/or caries risk at the earliest stages, we can better empower families to manage their children's oral health in concert with the professional office team and its efforts. When technology routinely allows us to inform parents of their child's risk level, as well as to inform them of the specific locations where early caries activity is occurring, it is certain that families will feel a greater sense of obligation to take part in preventing progression of disease.

**Figure 1:**
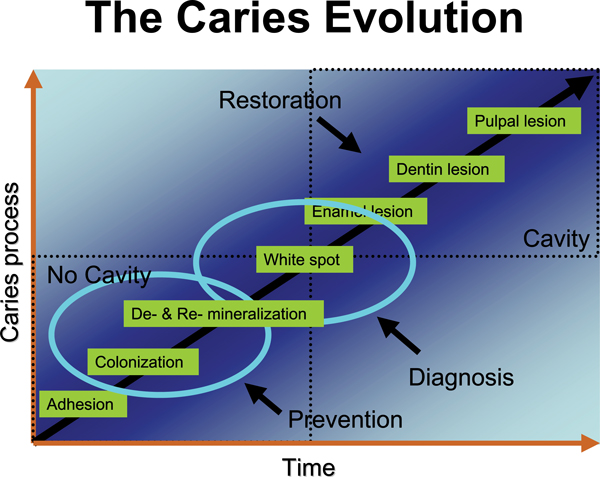
The Caries Evolution.

As we are able to detect the risk of caries and caries-specific lesions earlier, the dental industry will incorporate various new areas of scientific discovery to yield products along the continuum of development of caries (Figure 1). The "caries evolution" process notes various stages along the pathway of caries development where intervention products might be created. Any product developed to be used in the lower left quadrant of this chart provides a solution that is implemented prior to restorative dentistry. Given new discoveries in biofilm research, as well as in perfecting remineralization techniques, clearly there is significant product development opportunity. Only the compensation/reimbursement mechanisms will need to be put in place.

### Access to Care/the Silent Epidemic

In 2000, the Surgeon General of the United States, in the first ever Surgeon General's Report on Oral Health, [26] called dental caries in children "the silent epidemic." Included in this report was a detailed explanation of the access to care problem in this country. Eighty percent of dental disease occurs in 25% of the population, and it is this affected segment that least frequently has access to care. It is therefore essential that general practitioners and pediatric dentistry join forces in gaining access to care for all children. Given the growing number of courses, educational materials online, and efforts to train general dentists in the care of young children, we are all better enabled to bring children into our practices and to manage the solution to this problem.

## Conclusion

There are exciting times ahead in dentistry and, in particular, in caring for children. One of the features of this exciting present and future is the increased facility of managing caries as a disease, and not merely treating its results. It is clear that the years ahead will bring better and more powerful caries risk assessment tools and detection devices, all yielding more effective therapeutic interventions. How the marketplace will evolve to allow for these changes is unclear, but clearly the consumer/patient, dental professionals, and the dental pharmaceutical industry will play important roles. Just as the medical pharmaceutical business talks directly to the consumer to promote various drugs, so, too will dental professionals and the dental pharmaceutical industry. As opportunities become identified to create new drugs to deal with caries as a disease, treating it before surgical restorative intervention is needed; clearly the interaction of these three players in the marketplace will yield major changes in the dental industry as a whole.

## Competing interests

The author(s) declare that they have no competing interests.
